# Inguinal lymph node metastases from prostate cancer: clinical, pathology, and multimodality imaging considerations

**DOI:** 10.1590/0100-3984.2024.0013

**Published:** 2024-06-15

**Authors:** Sungmin Woo, Anton S. Becker, Soleen Ghafoor, Felipe de Galiza Barbosa, Yuki Arita, Hebert A. Vargas

**Affiliations:** 1 Department of Radiology, NYU Langone Health, New York, NY, USA; 2 Department of Radiology, Memorial Sloan Kettering Cancer Center, New York, NY, USA; 3 Institute of Diagnostic and Interventional Radiology, University Hospital Zurich, Zurich, Switzerland; 4 Department of Radiology, Hospital Sírio-Libanês, São Paulo, SP, Brazil; 5 Department of Radiology, Keio University School of Medicine, Tokyo, Japan

**Keywords:** Lymph nodes/pathology, Biopsy, Magnetic resonance imaging, Positron emission tomography computed tomography, Prostatic neoplasms/diagnostic imaging, Urethra/pathology, Linfonodos/patologia, Biópsia, Ressonância magnética, Tomografia por emissão de pósitrons combinada a tomografia computadorizada, Neoplasias da próstata/diagnóstico por imagem, Uretra/patologia

## Abstract

**Objective:**

To investigate clinical, pathology, and imaging findings associated with inguinal lymph node (LN) metastases in patients with prostate cancer (PCa).

**Materials and Methods:**

This was a retrospective single-center study of patients with PCa who underwent imaging and inguinal LN biopsy between 2000 and 2023. We assessed the following aspects on multimodality imaging: inguinal LN morphology; extrainguinal lymphadenopathy; the extent of primary and recurrent tumors; and non-nodal metastases. Imaging, clinical, and pathology features were compared between patients with and without metastatic inguinal LNs.

**Results:**

We evaluated 79 patients, of whom 38 (48.1%) had pathology-proven inguinal LN metastasis. Certain imaging aspects— short-axis diameter, prostate-specific membrane antigen uptake on positron-emission tomography, membranous urethra involvement by the tumor, extra-inguinal lymphadenopathy, and distant metastases—were associated with pathology-proven inguinal LN metastases (*p* < 0.01 for all). Associations with long-axis diameter, fatty hilum, laterality, and uptake of other tracers on positronemission tomography were not significant (*p* = 0.09–1.00). The patients with metastatic inguinal LNs had higher prostate-specific antigen levels and more commonly had castration-resistant PCa (*p* < 0.01), whereas age, histological grade, and treatment type were not significant factors (*p* = 0.07–0.37). None of the patients had inguinal LN metastasis in the absence of locally advanced disease with membranous urethra involvement or distant metastasis.

**Conclusion:**

Several imaging, clinical, and pathology features are associated with inguinal LN metastases in patients with PCa. Isolated metastasis to inguinal LNs is extremely rare and unlikely to occur in the absence of high-risk imaging, clinical, or pathology features.

## Abstract (pt)

**Objetivo:**

Investigar achados clinicopatológicos e de imagem associados a metástases linfonodais inguinais em pacientes com câncer de próstata (CaP).

**Materiais e Métodos:**

Estudo retrospectivo de uma única instituição de pacientes com CaP submetidos a exames de imagem e biópsia inguinal de linfonodos em 2000–2023. A imagem multimodalidade foi avaliada para morfologia inguinal do linfonodo, linfadenopatia fora da região inguinal, extensão do CaP primário/recorrente e sítios metastáticos não nodais. Características de imagem e clinicopatológicas foram comparadas entre pacientes com e sem linfonodos inguinais metastáticos pela patologia.

**Resultados:**

Entre 79 pacientes estudados, 38 (48,1%) apresentaram metástase inguinal de linfonodo comprovada patologicamente. Certos achados de imagem – diâmetro do eixo curto, captação do antígeno de membrana prostático específico na tomografia por emissão de pósitrons, envolvimento da uretra membranosa pelo tumor, linfadenopatia fora da região inguinal e metástases a distância – foram associados com metástases inguinais no linfonodo pela patologia (*p* < 0,01). Diâmetro de eixo longo, hilo gorduroso, lateralidade, captação em outros traçadores de tomografia por emissão de pósitrons não foram significativos (*p* = 0,09–1,00). Clinicopatologicamente, os pacientes com linfonodos inguinais metastáticos apresentaram maior antígeno prostático específico e foram mais resistentes à castração (*p* < 0,01); idade, grau histológico e tipo de tratamento não foram estatisticamente significantes (*p* = 0,07–0,37). Nenhum paciente apresentou metástase inguinal isolada no linfonodo na ausência de doença localmente avançada com envolvimento da uretra membranosa ou metástase a distância.

**Conclusão:**

Várias características de imagem e clinicopatológicas foram associadas a metástases em LNs inguinais em pacientes com CaP. A metástase isolada para os LNs inguinais é extremamente rara e é improvável que ocorra na ausência de características de imagem e clinicopatológicas de alto risco.

## INTRODUCTION

Prostate cancer (PCa) most commonly spreads to bones and lymph nodes (LNs) in the pelvis and retroperitoneum^(1,2)^. Although inguinal LN metastases from PCa are considered rare, the true prevalence is unknown. Jackson et al.^(3)^ found that 5 (9.1%) of 55 men with PCa presenting with retroperitoneal or pelvic adenopathy at baseline had enlarged inguinal LNs that were enlarged (short-axis diameter ≥ 0.8 cm), although there was no verification by pathology. Recently, Schiller et al.^(4)^ investigated 799 LNs in 233 patients who underwent prostatespecific membrane antigen positron-emission tomography/computed tomography (PSMA PET/CT) and found that 10 inguinal LNs (1.3%) were suspicious (i.e., “PET-positive”), although there was again no verification by pathology. The remainder of the literature consists of a few case reports in which investigators raise hypotheses on potential pathways for the spread to inguinal LNs, such as altered lymphatic drainage after prostatectomy^(5,6,7,8,9,10,11,12,13)^. In clinical practice, questions regarding abnormal inguinal LNs raising concern for metastases are becoming increasingly common, especially with the growing popularity of molecular imaging (e.g., PSMA PET/CT). Hence, there is an unmet clinical need for better understanding of this atypical pattern of spread, in terms of its prevalence and predictive factors, with pathology as the ground truth^(14)^. A better understanding of these factors may lead to improved patient care, given that inguinal LNs would not be included in conventional radiation therapy (RT) fields nor in extended pelvic LN dissection templates.

The objective of this study was to identify predictive findings for inguinal LN metastases from PCa. To that end, we investigated clinical, pathology, and multimodality imaging findings in patients with PCa who underwent inguinal LN biopsy.

## MATERIALS AND METHODS

### Study population

**Figure 1.**
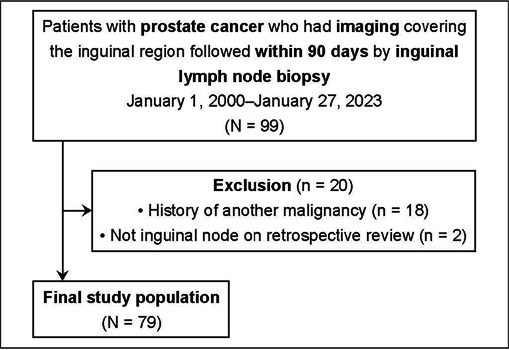
Flow chart of the patient selection process.

This was a retrospective single-center study based on a review of electronic medical records. The institutional review board waived the requirement for informed consent, and the study was conducted in accordance with Health Insurance Portability and Accountability Act guidelines. The inclusion criteria were as follows: having been diagnosed with PCa; and images covering the inguinal region having been obtained within 90 days of the inguinal LN biopsy. The data evaluated were related to the period from January 1, 2000 to January 27, 2023. Patients with a known diagnosis of another malignancy commonly associated with inguinal LN metastasis (e.g., lymphoma, melanoma, anal cancer, penile cancer, etc.) were excluded, as were those in whom the biopsied LN was not an inguinal LN. The patient selection process is shown in Figure 1.

### Imaging assessments

We assessed images obtained with all available modalities, including CT, magnetic resonance imaging (MRI), bone scintigraphy, and PET/CT. There was variability arising from the use of different scanners, protocols, and technical parameters. However, almost all CT and MRI scans included contrast-enhanced images and all MRI scans were performed with standard sequences.

Images were interpreted in consensus by two genitourinary radiologists with 3 and 12 years of post-fellowship experience, respectively, who were aware of the diagnosis of PCa but were blinded to all other clinical data and pathology findings. The following aspects were assessed: various morphologic aspects of the inguinal LN; the extent of primary or recurrent PCa; the presence and distribution of lymphadenopathy outside the inguinal region; and other sites of metastasis. When there was more than one enlarged inguinal LN, the largest and most suspicious LN was evaluated as representative of the patient. First, on all CT and MRI studies, we documented LN size (determined by bidimensional measurement), the presence of a fatty hilum in the LN, and laterality of the LNs. We also documented the type of radiotracer used on PET/CT—PSMA, choline, fluciclovine, or fluorodeoxyglucose (FDG)—and its uptake, recorded as the maximum standardized uptake value (SUVmax). If no diagnostic CT scan was available but there was an available PET/CT scan, the correlative CT component of the PET/CT scan was used for assessing morphological and location-related features. Second, if a dedicated MRI study with a prostate protocol was available, we evaluated whether there was involvement of the membranous urethra—either by the primary tumor involving the apex or by a recurrent tumor involving the vesicourethral anastomosis. The rationale for this was that the more distal portions of the urethra (typically the penile portion and potentially the membranous portion) are known to drain to inguinal LNs^(15)^. Third, we categorized the location of lymphadenopathy into the following categories: unilateral inguinal-only; bilateral inguinal-only; inguinal with pelvic or retroperitoneal involvement; and diffuse (above and below the diaphragm). Fourth, we documented the sites of non-nodal metastases using all available imaging modalities and categorized them as follows: none; bone-only; visceral organs; and atypical sites with or without bones. For the purpose of this study, lymphadenopathy and metastases were defined per conventional definitions in the literature^(16,17,18,19,20)^; for example, lymphadenopathy was defined as either a short-axis diameter > 1.0 cm or radiotracer uptake above the respective background tissue avidity. Finally, we assessed images to look for any explanation for reactive enlargement of inguinal LNs (e.g., inguinal hernia repair mesh, lymphocele, etc.).

### Clinical data and pathology findings

The electronic medical records were searched to identify the following information: age; International Society of Urogenital Pathology (ISUP) grade group; serum prostatespecific antigen (PSA) levels at baseline and at recurrence; treatment status (treatment-naïve vs. previously treated); the type (if any) of initial treatments (e.g., radical prostatectomy [RP], RT, androgen-deprivation therapy, etc.); the interval from RP or RT to imaging and from imaging to biopsy; castration status (sensitive vs. resistant); pathology on inguinal LN biopsy; and last follow-up data.

### Statistical analysis

To compare clinical, pathology, and imaging variables between the inguinal LNs that were confirmed as metastatic on biopsy and those that were not, we used Wilcoxon rank-sum test for continuous variables and chi-square test or Fisher’s exact test for categorical variables. Statistical analysis was performed with R (version 4.3.0, R Project for Statistical Computing, Vienna, Austria). Values of *p* < 0.05 were considered significant.

## RESULTS

### Clinical data and pathology findings

Table 1 summarizes the clinical data and pathology findings related to the patients evaluated. In brief, there were 79 patients with median age of 67 years (interquartile range [IQR]: 62–75 years). The ISUP grade group at baseline was ≥ 3 in 54 (68.4%) of those patients. Twenty-seven patients (34.2%) were treatment-naïve, and 52 (65.8%) had been previously treated. Twenty-one patients (26.6%) had castration-resistant PCa. A median of two (IQR: 1–3) imaging modalities were used in order to assess inguinal LNs and metastatic disease, most commonly CT, in 78 patients (98.7%); prostate MRI, in 30 (38.0%); bone scan, in 26 (32.9%); and PSMA PET/CT, in 18 (22.8%). Sixty-two (78.5%) of the patients were assessed with two or more imaging modalities. Core biopsies, guided by ultrasound or CT, revealed metastatic inguinal LNs on pathology in 38 (48.1%) of the 79 patients.

**Table 1:** Clinical and pathology characteristics of patients with PCa who underwent inguinal LN biopsy.

Characteristic	(N = 79)*
Age (years), median (IQR)	67 (62–75)
ISUP grade group†	
1	6 (8.0)
2	15 (20.0)
3	18 (24.0)
4	14 (18.7)
5	22 (29.3)
PSA (ng/mL), median (IQR)	
At baseline	10.0 (6.3–27.0)
At suspected recurrence	2.8 (0.6–23.9)
Initial treatment	
Treatment-naïve	27 (34.2)
RP	38 (48.1)
RT	9 (11.4)
Systemic	5 (6.3)
Castration status	
Resistant	21 (26.6)
Sensitive	58 (73.4)
Inguinal LN metastasis	38 (48.1)
Interval (days), median (IQR)	
Imaging to biopsy	20 (12–32)
Initial treatment to suspected recurrence	2,329 (699–4,548)
Biopsy to last follow-up	1,100 (343–1,755)
Imaging modalities used to assess inguinal LNs and other aspect	
CT‡	78 (98.7)
Prostate MRI	30 (38.0)
Whole-body MRI	2 (2.5)
Bone scan	27 (34.8)
PSMA PET/CT	18 (22.7)
Choline PET/CT	6 (7.6)
Fluciclovine PET/CT	4 (5.1)
FDG PET/CT	13 (16.5)

* Data presented as n (%), except where otherwise indicated.

† For four patients (all with metastatic inguinal LNs), no data were available, because the diagnosis of PCa was confirmed through analysis of an inguinal LN biopsy specimen.

‡ In one patient, no CT scan was available and metastatic disease was evaluated with whole-body MRI.

### Association of imaging, clinical, and pathology features with inguinal LN metastasis

Table 2 shows the association between imaging features and inguinal LN metastasis. In their short-axis diameter, metastatic inguinal LNs were larger than were non-metastatic LNs (1.7 cm, IQR: 1.3–2.2 vs. 1.2 cm, IQR: 0.9–1.6; *p* < 0.01), although there was no significant difference in long-axis diameters (2.2 cm, IQR: 1.7–3.0 vs. 1.9 cm, IQR: 1.4–2.4; *p* = 0.09). There were no significant differences between those two groups in terms of the presence of a fatty hilum (*p* = 0.22) or in terms of laterality (*p* = 0.23). On PSMA PET/CT, radiotracer uptake was greater in metastatic LNs (SUV_max_ = 19.5, IQR: 14.6–25.1 vs. SUVmax = 2.2, IQR: 1.8–3.0). However, no differences were seen on choline, fluciclovine, or FDG PET/CT (*p* = 0.17–1.00).

**Table 2:** Associations of inguinal LN metastasis with imaging features and with clinical/pathology features.

				Inguinal LN status		*P*-value
	Characteristic			Metastatic (n = 38)	Nonmetastatic (n = 41)	
Imaging	Inguinal LN	Size (cm)	Long-axis diameter	2.2 (1.7–3.0)	1.9 (1.4–2.4)	0.094
			Short-axis diameter	1.7 (1.3–2.2)	1.2 (0.9–1.6)	0.002
		Fatty hilum		8 (23.5)	15 (36.6)	0.213
		Laterality	Left	19 (50.0)	26 (63.4)	0.229
			Right	19 (50.0)	15 (36.6)	
		Radiotracer uptake (SUVmax)*	PSMA	19.5 (14.6–25.1)	2.2 (1.8–3.0)	< 0.001
			Choline	3.4 (3.4–3.4)	3.0 (2.9–4.4)	> 0.999
			Fluciclovine	16.3 (16.3–16.3)	3.6 (3.2–3.7)	0.500
			FDG	8.4 (8.2–14.3)	6.5 (4.8–11.7)	0 .174
	Membranous urethra involvement by primary tumor†		All patients	9/12 (75.0)	1/18 (5.6)	< 0.001
			Baseline	6/7 (85.7)	1/7 (14.3)	0.029
			Post-RP	3/5 (60.0)	0/11 (0.0)	0.018
	Distribution of lymphadenopathy		Unilateral inguinal	2 (5.3)	21 (51.2)	< 0.001
			Bilateral inguinal	0 (0.0)	14 (34.1)	
			Inguinal + pelvic or retroperitoneal	28 (73.7)	1 (2.4)	
			Inguinal + diffuse	8 (21.1)	5 (12.2)	
	Sites of non-nodal metastases		None	17 (44.7)	39 (95.1)	< 0.001
			Bone	15 (39.5)	2 (4.9)	
			Visceral‡	6 (15.8)	0 (0.0)	
	Reactivity explained			0 (0.0)	7 (17.1)	0.012
Clinical/Pathology	Age (years)			68 (62–76)	67 (60–72)	0.370
	ISUP grade group§		1	1 (2.9)	5 (12.2)	0.072
			2	4 (11.8)	11 (26.8)	
			3	8 (23.5)	10 (24.4)	
			4	6 (17.6)	8 (19.5)	
			5	15 (44.1)	7 (17.1)	
	PSA level at baseline (ng/mL)			13.8 (7.2–41.3)	9.1 (5.7–13.6)	0.027
	PSA level at recurrence (ng/mL)			23.0 (7.4–113.9)	0.6 (0.2–2.6)	< 0.001
	Initial treatment		Baseline	11 (28.9)	16 (39.0)	0.110
			RP	16 (42.1)	22 (53.7)	
			RT	7 (18.4)	2 (4.9)	
			Systemic	4 (10.5)	1 (2.4)	
	Castration status		Resistant	19 (50.0)	2 (4.9)	< 0.001
			Sensitive	19 (50.0)	39 (95.1)	
	Days from initial treatment to suspected recurrence¶			3,881 (2585–6287)	737 (272–2272)	< 0.001

Continuous variables are presented as median (IQR); and categorical variables are presented as n (%).

* Patient subsamples, by type of radiotracer used for PET/CT: PSMA (n = 18; 8 and 10 patients with and without metastatic inguinal LNs, respectively); choline (n = 6; 1 and 5 patients with and without metastatic inguinal LNs, respectively); fluciclovine (n = 4; 1 and 3 patients with and without metastatic inguinal LNs respectively); and FDG (n = 13; 7 and 6 patients with and without metastatic inguinal LNs, respectively)

† Analyzed for patients that underwent dedicated prostate MRI (n = 30; 12 and 18 patients with and without metastatic inguinal LNs, respectively), 14 scans having been performed for baseline assessment (7 and 7 patients with and without metastatic inguinal LNs, respectively), and 16 having been performed after RP (5 and 11 patients with and without metastatic inguinal LNs, respectively)

‡ With or without bone metastases

§ For four of the 38 patients with metastatic inguinal LNs, the ISUP grade group was not assessed, because the diagnosis of PCa was confirmed through analysis of an inguinal LN biopsy specimen

¶ Analyzed for 46 patients that had previously been treated with RP or RT

**Figure 2.**
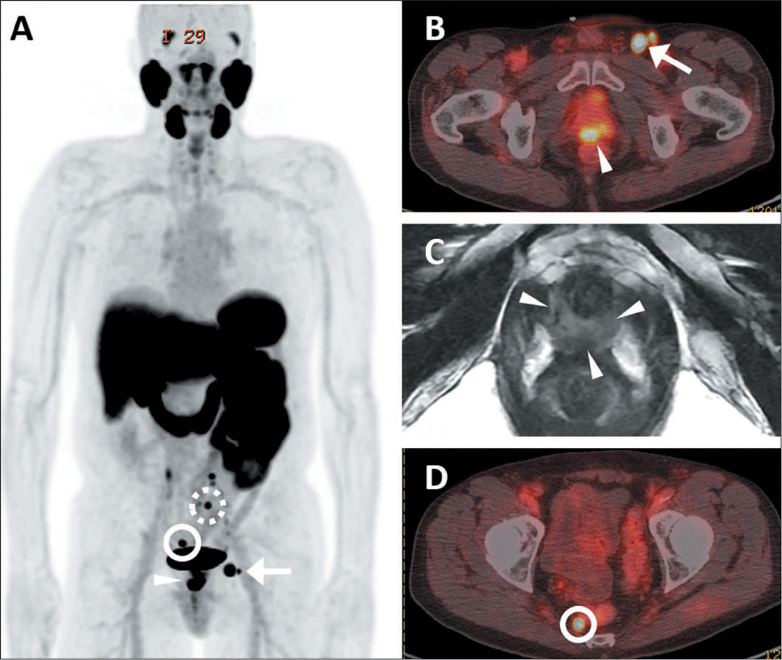
PSMA PET/CT and prostate MRI of a 66-year-old man with newly diagnosed ISUP grade group 5 PCa. PSA was 18.2 ng/mL at baseline. **A:** Maximumintensity projection PSMA PET/CT image showing a few enlarged and radiotracer-avid pelvic and left inguinal LNs, as well as the primary prostate tumor. **B:** Axial fused PSMA PET/CT shows the biopsied left inguinal LN (arrow) measuring 1.2 **×** 1.0 cm with an SUVmax of 27.8. **C:** Prostate MRI showing an apically located primary prostate tumor (arrowheads) encasing the membranous urethra and in contact with lower anterior rectum. **D:** Axial fused PSMA PET/CT showing a metastatic right mesorectal LN (solid circle) together with a superior rectal LN (broken circle in **A**). No suspicious pelvic LNs were noted at the typical sites (e.g., external, internal, common iliac, retroperitoneal, etc.).

Among the 30 patients who underwent prostate MRI, membranous urethra involvement (Figure 2) was seen in 9 (75.0%) of the 12 metastatic inguinal LNs, compared with only 1 (5.6%) of the 18 that were nonmetastatic. That was consistently shown, in the 14 patients evaluated at baseline (85.7% vs. 14.3%; *p* = 0.03) and in the 16 patients evaluated post-RP (60.0% vs. 0.0%; *p* = 0.02).

The distribution of lymphadenopathy differed significantly between the patients with metastatic inguinal LNs and those with nonmetastatic inguinal LNs (*p* < 0.01). Of the 38 patients with metastatic inguinal LNs, 28 (73.7%) had concurrent pelvic/retroperitoneal lymphadenopathy. Among the 41 patients with nonmetastatic inguinal LNs, lymphadenopathy was unilateral in 21 (51.2%) and bilateral in 14 (34.1%), being restricted to the inguinal region in both cases. There was no substantial difference between the two groups in terms of the proportion of patients with diffuse lymphadenopathy (21.1% vs. 12.2%).

**Figure 3.**
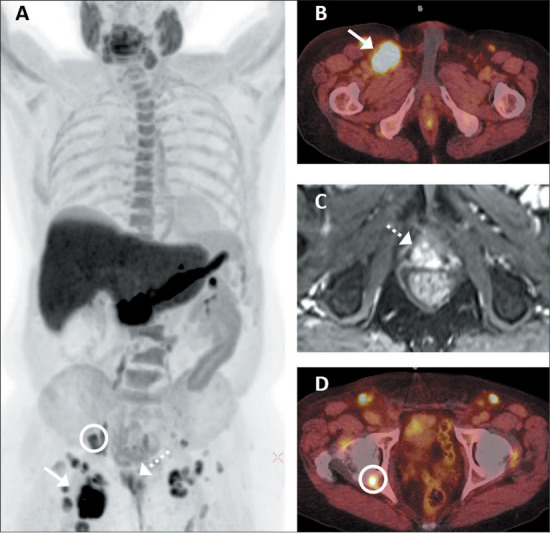
Fluciclovine PET/CT and prostate MRI of an 81-year-old man with ISUP grade group 4 PCa who underwent RT followed by androgen-deprivation therapy and multiple systemic treatments, the tumor subsequently becoming castration-resistant. PSA was 7.0 and 0.7 ng/mL at baseline and at the time of imaging, respectively. **A:** Maximum-intensity projection fluciclovine PET/CT image showing numerous enlarged and radiotracer-avid bilateral inguinal, pelvic, and lower retroperitoneal LNs. **B:** Axial fused PET/CT showing the biopsied right inguinal LN (solid arrow) measuring 5.1 **×** 4.5 cm with an SUVmax of 16.3. **C:** Prostate MRI showing a locally recurrent mass (broken arrow) involving vesicourethral anastomosis. **D:** Axial fused PET/CT at a different level showing fluciclovine-avid bone metastasis at the right ischium (circle). Biopsy revealed a metastatic inguinal LN.

**Figure 4.**
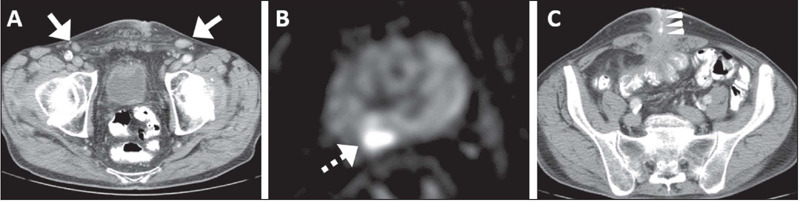
CT and prostate MRI of a 67-year-old man with newly diagnosed ISUP grade group 4 PCa. PSA was 19.2 ng/mL at baseline. **A:** Axial CT showing bilateral enlarged inguinal LNs, including the biopsied one on the left (arrow). **B:** Prostate MRI with diffusion-weighted imaging showing the dominant lesion (broken arrow) in the right posterior peripheral zone, not extending to the apex. **C:** Axial CT showing an enterocutaneous fistula related to known Crohn’s disease and demonstrating the enteric passage of contrast media (arrowheads) through the anterior abdominal wall (a potential cause of the reactive lymphadenopathy). Inguinal LN biopsy was negative for cancer. The patient was subsequently treated with brachytherapy and is free of recurrence at 368 days after diagnosis.

Bone metastases (Figure 3) were seen in 15 (39.5%) of the 38 patients with metastatic inguinal LNs, compared with only one (4.9%) of the 41 patients without metastatic inguinal LNs (*p* < 0.01). Finally, while possible explanations for reactive inguinal LNs, such as enterocutaneous fistula (Figure 4), were seen in 7 (17.1%) of the patients without metastatic inguinal LNs, although no alternative explanation was identified in the patients with metastases.

Table 2 shows the association that clinical features and pathology findings had with inguinal LN metastasis. Patients with metastatic inguinal LNs had significantly higher PSA levels at baseline (*p* = 0.03) and at recurrence (*p* < 0.01), as well as more commonly having castration-resistant PCa (*p* < 0.01) and having had a longer time from RP or RT to imaging (*p* < 0.001). There was no significant difference between the patients with and without inguinal LN metastasis in terms of age (*p* = 0.37), grade group (*p* = 0.07), or initial treatment (*p* = 0.11).

### Inguinal-only lymphadenopathy

**Figure 5.**
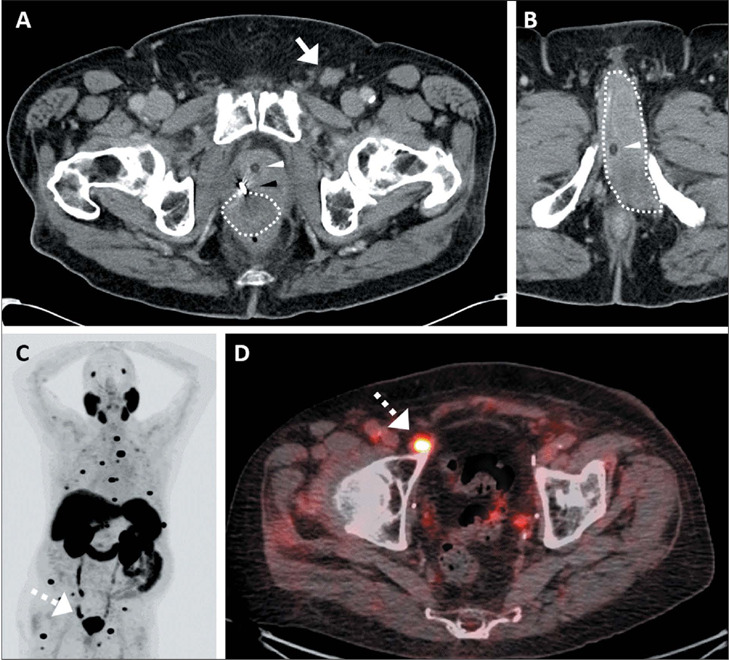
Two patients with PCa and biopsy-proven inguinal LN metastases presenting with unilateral inguinal lymphadenopathy. **A,B:** CT of a 90-year-old man with ISUP grade group 4 PCa treated with RT 11 years prior. PSA was 13.0 ng/mL at baseline, with a biochemical response, now rising at 22.3 ng/mL. **A:** CT showing a left inguinal LN measuring 2.0 **×** 1.6 cm. Note the fiducial marker (black arrowhead), Foley catheter (white arrowhead), and rectal invasion by the superior portion of a recurrent prostate tumor (dotted outline). **B:** At a lower level, a recurrent prostate tumor (dotted outline) is shown directly invading the left corpus spongiosum of the penis through the membranous urethra and lower rectum. **C,D:** PSMA PET/CT of an 83-year-old man with ISUP grade group 3 PCa treated 17 years prior with RP followed by salvage RT and multiple lines of systemic treatment, the tumor subsequently becoming castration-resistant, with a PSA of 1.5 ng/mL. **C:** Maximumintensity projection PSMA PET/CT image showing widespread radiotracer-avid bone metastases. **D:** Axial fused PSMA PET/CT shows new deep right inguinal LN (broken arrow), measuring 1.1 **×** 1.1 cm, with an SUVmax of 17.8.

Of the 79 patients evaluated, only two (2.5%) had inguinal-only lymphadenopathy (i.e., no lymphadenopathy in the pelvis, retroperitoneum, or elsewhere), and both of those patients had metastatic inguinal LNs confirmed on pathology (Figure 5). Both had high-grade PCa (grade group ≥ 3), had received treatment of the primary tumor, and had a high PSA level either at baseline or at recurrence. One had a large recurrent prostate mass invading the lower rectum and extending via the membranous urethra into the corpus cavernosum. The other had concurrent widespread bone metastases. In essence, no patient had isolated inguinal LN metastasis in the absence of coexisting non-inguinal nodal metastases, bone metastasis, or membranous urethra involvement by the prostate tumor on multimodality imaging.

### Details of nonmetastatic inguinal LNs

**Figure 6.**
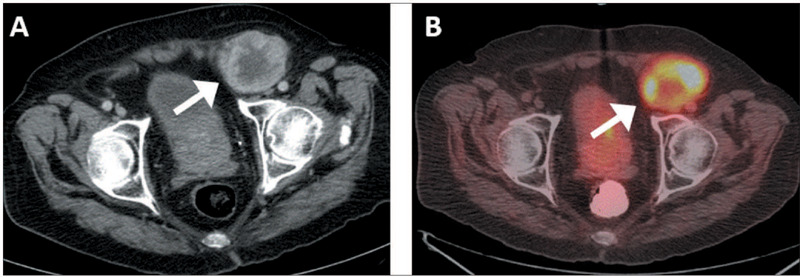
CT and FDG PET/CT of an 82-year-old man with ISUP grade group 1 PCa treated with RT six years prior. PSA was 11.7 ng/mL at baseline, with a biochemical response, now rising at 2.5 ng/mL. **A:** Contrast-enhanced axial CT showing an enlarged, centrally necrotic left inguinal LN (arrow) measuring 7.3 × 5.0 cm. **B:** Fused axial FDG PET/CT showing a radiotracer-avid inguinal LN (arrow) with an SUVmax of 15.1. There were no findings suspicious for recurrence or metastases. Biopsy confirmed liposarcoma, which was suspected to be related to prior RT.

**Figure 7.**
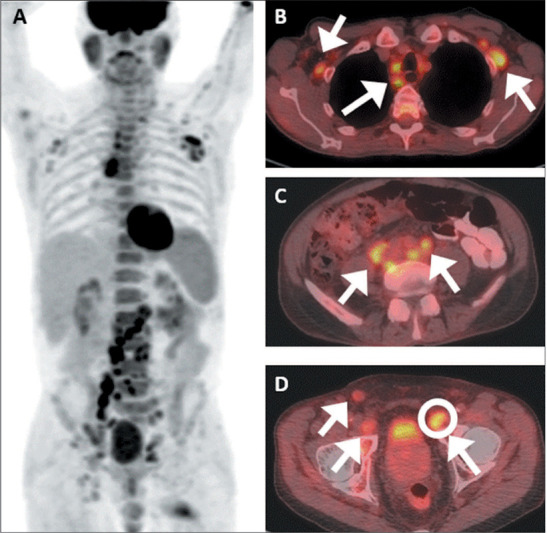
FDG PET/CT of a 73-year-old man with newly diagnosed ISUP grade group 3 PCa. PSA was 6.3 ng/mL at baseline. **A:** Maximum-intensity projection FDG PET/CT image showing numerous radiotracer-avid LNs with diffuse distribution. **B–D:** Fused axial FDG PET/CT scans showing widespread lymphadenopathy (arrows) involving the thoracic, retroperitoneal, pelvic, and bilateral inguinal nodal stations. **D:** Left deep inguinal LN (circle) measuring 2.9 **×** 2.6 cm, with an SUVmax of 4.6 was biopsied and was initially negative for malignancy. Additional biopsy of a retroperitoneal LN revealed angioimmunoblastic T-cell lymphoma.

Among the 41 patients whose inguinal LN biopsy results were negative for PCa, nonprostatic malignancy in an inguinal LN was confirmed in five (12.2%), including liposarcoma, which was presumed to be radiation-associated, in one (Figure 6); chronic lymphocytic leukemia/ small lymphocytic lymphoma, in two; and malignant peripheral nerve sheath tumor, in one. In one of those patients, a nonviable necrotic tumor was found in the inguinal LN specimen and diffuse large B-cell lymphoma was subsequently identified on biopsies of obturator and external iliac LNs. In two other patients, reactive changes were seen in the inguinal LN specimen but pelvic LN dissection performed concurrently with RP (Figure 7) revealed lymphoma involvement (angioimmunoblastic T-cell lymphoma in one patient and mantle cell lymphoma in the other). Of these seven patients, four had inguinalonly lymphadenopathy, three had diffuse lymphadenopathy, and none had concurrent distant metastases. In the remaining patients, follow-up after biopsy for a median of 1,115 days (IQR, 368–1,826 days) did not raise suspicion of inguinal LN malignancy (related or unrelated to PCa).

## DISCUSSION

This study assessed multimodality imaging findings, as well as clinical and pathology features, in patients with PCa who underwent inguinal LN biopsy. We specifically addressed the unmet clinical need to improve understanding of the rare phenomenon of PCa spread to inguinal LNs. Historically, the reported prevalence of this phenomenon has been low (1.3–9.1%), with the caveat that this is based on imaging and lack of pathological proof^(3,4)^. Focusing on a specific scenario in which patients with PCa underwent inguinal LN biopsy provided us with a robust, pathology-based reference standard and allowed us to identify a high (48.1%) prevalence of inguinal LN metastasis among patients with PCa. Because metastasis to inguinal LNs would not be covered under conventional RT or LN dissection templates, better ability to predict the presence of inguinal LN metastasis will assist in stratifying a specific management plan for patients with PCa, potentially avoiding unnecessary biopsies. These findings could be useful for improving patient care.

Several imaging features differed between metastatic and nonmetastatic inguinal LNs. Metastatic inguinal LNs were larger (only in their short-axis diameters) than were nonmetastatic LNs, which is in agreement with a recently proposed Node Reporting and Data System that defines the “normal” size of an inguinal LN as a short-axis diameter < 1.5 cm^(16)^. However, size cannot be used as the sole criteria, given that there was substantial overlap and that microscopic metastases are undetectable by CT and MRI^(21)^. Radiotracer uptake on PSMA PET/CT was greater in metastatic inguinal LNs than in those that were nonmetastatic. That is indicative of the powerful capability of PSMA PET/CT to detect and localize metastatic disease^(22)^. Nevertheless, caution is warranted because the pretest likelihood of inguinal LN metastasis is low, only 18 (22.8%) of the patients in our sample underwent PSMA PET/CT, and PSMA uptake in inguinal LNs can be attributable to other malignancies, such as penile cancer and lymphoma^(4,23,24)^.

In the present study, an MRI finding of membranous urethra involvement by the tumor was significantly associated with inguinal LN metastasis, in pre- and post-treatment settings. That is likely related to anatomical factors, such as lymphatic drainage. Although the prostatic urethra typically drains to obturator and external iliac LNs and the penile urethra typically drains to superficial inguinal LNs, the membranous portion may have variable drainage to either^(15,25)^. To our knowledge, this has been described in only two case reports: one in which the patient presented with a penile nodule and inguinal lymphadenopathy^(6)^; and another in which the patient had a primary tumor extending diffusely into the corpus spongiosum with inguinal LN metastasis^(11)^. Because MRI is the standard-of-care imaging modality for PCa in the pre-treatment setting or when there is suspicion of recurrence, radiologists should remember to scrutinize the membranous urethra (or any region that may drain to the inguinal LNs, such as the lower rectum, penis, and perineum) when inguinal lymphadenopathy is encountered, especially in apical tumors with extraprostatic extension^(26,27)^.

In our patient sample, the distribution of lymphadenopathy and the sites of metastases on imaging were significant factors associated with inguinal LN metastasis. Concurrent imaging evidence of well-known patterns of LN metastases (i.e., pelvic or retroperitoneal) and bone metastases were also associated with metastatic inguinal LNs. Only one patient had an isolated inguinal LN metastasis without coexisting extra-inguinal lymphadenopathy or bone metastasis—that patient had membranous urethra involvement by the prostate tumor. That adds pathology confirmation to a few prior reports without biopsy correlation, showing that inguinal LN metastases are typically accompanied by widespread metastases in other LNs and bone^(8,9,12)^. However, caution is needed in patients with diffuse lymphadenopathy, which can represent either widespread LN metastases or lymphoma. With regards to isolated enlarged inguinal LNs, most of which were nonmetastatic in our sample, imaging may reveal reasons for the reactive changes.

Clinical and pathology findings were also found to be important in our study. As previously stated, the patients with metastatic inguinal LNs had higher PSA levels at baseline and at recurrence. That is likely a clinical reflection of the imaging findings mentioned above (concurrent pelvic or retroperitoneal lymphadenopathy and bone metastases). The primary tumor grade group did not differ significantly between the patients with and without metastatic inguinal LNs. We speculate that although higher grade tumors are more likely to metastasize, that does not necessarily mean that they will spread to atypical locations. Inguinal LN metastases were seen throughout grade groups 1–5 in our study, as has been described in case reports^(5,6,7,8,9,10,11,12,13)^. In two cohorts of patients with predominantly high-grade group PCa who underwent dissection of pelvic LNs, metastasis in deep inguinal LNs was confirmed on pathology in only one (2.6%) and three (1.1%) of the 39 and 285 patients in each cohort, respectively^(28,29)^. It has been suggested, on the basis of anecdotal evidence, that altered lymphatic drainage following prostatectomy creates a potential pathway for spread to the inguinal LNs. However, in the present study, we found no association between prior treatment and inguinal LN metastasis. In fact, approximately a third of the patients presented with inguinal LN metastasis at baseline. In addition, castration resistance and longer time from RP or RT to recurrence, both of which likely reflect a greater number and variety of treatments, were associated with greater prevalence of inguinal LN metastasis.

Our study has some limitations. First, it has all of the limitations inherent to a retrospective single-center study with a small patient sample. Nevertheless, to our knowledge, ours was the largest cohort to date in a study investigating inguinal LNs in patients with PCa. In addition, the fact that the sample was restricted to patients who underwent inguinal LN biopsy could raise concern for a selection bias (skewing toward a higher-risk patient sample, given that inguinal LN biopsy is not routinely performed in all patients with PCa) and undersampling. However, histopathological evaluation was necessary, not only to determine the metastatic status of the inguinal LN but also to provide additional details of nonprostatic malignancies. Furthermore, in order to mitigate the risk of undersampling, we ensured that the follow-up period was sufficient for patients in whom biopsy indicated nonmalignant status. Moreover, multivariate analysis to determine the impact of each of the imaging clinical, and pathology variables was not feasible, especially because certain analyses involved only a small subset of patients (e.g., membranous urethra invasion was assessed only in the 30 patients who underwent prostate MRI).

## CONCLUSIONS

Several imaging, clinical, and pathology features appear to be associated with biopsy-proven inguinal LN metastasis in patients with PCa. Isolated metastasis to an inguinal LN from a prostatic tumor is extremely rare and is unlikely to occur in the absence of high-risk clinical and imaging features such as high PSA, locally advanced disease, and coexisting metastasis.
